# Progress in Medicine

**Published:** 1899-09-23

**Authors:** 


### Progress in Medicine.

### PÆDIATRICS.

(*Continued from page* 422.)

**Treatment of Chorea by Sulphocarbolate of Soda.**—A case of severe chorea successfully treated by large doses of sulphocarbolate of soda, has been recorded by Dr. Stacey Wilson.[7] He refers to the serious nature of severe chorea, and the tendency to death from exhaustion, and states that in his own experience no treatment has seemed to have any effect. In the case reported, that of a girl aged 22 years in a third attack, all the symptoms of severe chorea were present, along with dryness and roughness of the skin, blueness and coldness of the hands, and a scattered red capillary flush. All other treatment having proved useless, on the eleventh day after admission to hospital he ordered the patient 20 grs. of sodium sulphocarbolate every two hours and 1 gr. of quinine every two hours, to be taken alternately. For occasional use 30 gr. doses of chloral were prescribed. After thirty-six hours most marked improvement set in, and "by the end of the fourth day she had become quite sensible and very much quieter, and was sleeping the greater part of the day and night. The patient made a speedy and satisfactory recovery." He suggests that many cases of chorea may be due to micro-organisms in the blood, and that thus the action of the sulphocarbolates is explained. He advises also a full and stimulating diet, of which uncooked or partially pancreatised raw meat juice is an important part.

**Empyema of the Thorax.**—Dr. J. B. Bogart[8] has recorded his experience in 23 cases of this affection surgically treated. The left side was involved 14 times, the right side seven times, and both sides twice. He strongly recommends a preliminary aspiration in all cases of large effusion with dyspnœa, and on the following day, or two days later, resection, under general anæsthesia, of a portion of the ninth rib in the posterior axillary line. If the amount of fluid is small and the symptoms are not urgent aspiration may be dispensed with. He points out that if an empyema is not relieved by operation, spontaneous evacuation may be anticipated in from two to three months after the onset of pulmonary symptoms, but this rarely results in the cure of the disease. Large drainage tubes, one or more, were employed, and were not removed until the cavity had been obliterated by the expansion of the lung and the discharge had ceased.

**Night Terrors.**—A very graphic account of this affection and associated disorders in children comes from the pen of Dr. Leonard Guthrie.[9] He accepts the two classes into which they have been divided by Silberman, namely, idiopathic and symptomatic, but thinks that a reflex source of irritation, which is the determining cause in the latter class, may also exist in the former although unrecognised. No valid distinction can be made between night terrors and nightmares, between seeing visions and merely dreaming dreams. "Little children are frightened out of their wits at hallucinations because they do not know what they are. Older children see them and are terrified by them, but usually will not admit it for fear of being laughed at, or accused of having over-eaten themselves. Adults see them, and send an embroidered account of the vision to the Psychological Research Society." Amongst the exciting causes of night terrors he enumerates aural vertigo, night sweats, febrile disturbance, actual pain, intestinal worms, and gastro-intestinal affections. To the last of these too much prominence is sometimes given, and the writer is more inclined to ascribe both the night terrors and the dyspepsia to a common cerebral origin. In idiopathic cases the primary cause seems to be in the neurotic temperament of the patient, and attention must be directed in the treatment to the patient's surroundings, home and school life, and companions. The presence of an ignorant and cruel nurse, or the teasing and punishments and lessons of school life may produce night terrors in a neurotic child sufficient to warp his whole life. Medicinally, the author recommends paraldehyde or bromides for a few nights in symptomatic cases, with removal of the exciting cause; and, in the idiopathic variety, a course of mixed bromides of soda, potash, and ammonium, which may have to be continued for a considerable period.

From a study of 32 cases of night terrors in children, Dr. Rey[10] concludes that adenoid hypertrophy is the most frequent cause, producing a carbonic acid poisoning through interference with the respiration. In all of his cases the adenoid growths were removed and the night terrors ceased.

**The Treatment of Enuresis by Distension.**—For cases of incontinence of urine which persist after puberty this treatment has been found effective. The theory is that the condition may be present in persons otherwise healthy, owing to a diminished capacity of the bladder. Dr. Haven[11] has found the treatment successful in two cases of long-standing enuresis, his patients being girls aged 17 and 18 years. The bladder was injected daily with a 4 per cent. solution of boric acid to the point of discomfort. At first only eight ounces could be tolerated, but after six weeks 20 ounces could be introduced, and the girl was completely cured. A similar result followed in the second case.

**Thyroid Treatment for Backward Children.**—The success of this treatment in connection with cretinism has led to its extended employment in many other forms of imbecility and delayed development. Dr. H. H. Vinke[12] gives the result of his experience in three cases. A female child, three years old, the daughter of a goitrous mother, was dull, stupid, unable to talk, and possessed of an exaggerated fear of strangers. The result of treatment by thyroid gland was that heavy layers of fat, irregularly distributed in thick ridges over the body, were considerably diminished in size. Another case is that of a girl, three years old, stunted, unable to walk or talk, with myxœdematous deposits on the dorsal surfaces of both feet. Under thyroid treatment for a year she had learned to walk and talk, had gained quite an intelligent look, and had lost the fatty swellings on the feet. A third case is that of a macrocephalic idiot, five years old, who under thyroid treatment showed increased intelligence and grew two inches, but had not learned to talk. Dr. Vinke believes that in future thyroid feeding will have a prominent place in the treatment of all forms of idiocy, and will prove valuable in improving the mental and physical condition of these degenerates.

**The Plantar Reflex in Infants.**—In 1898 Babinsky pointed out that whereas in adults flexion of the toes was the normal response obtained on stimulating the sole of the foot, extension of the toes results in infants. This difference is of importance because in many nervous lesions the plantar reflex is altered, and instead of being one of flexion is found to be one of extension, resembling the infantile type. This subject has been further investigated by Dr. James Collier,[13] who finds that the "infantile response" to irritation of the sole occurs first as extension of the great toe, followed by extension and spreading out of all the toes, with eversion of the foot and dorsiflexion of the ankle, and subsequently flexion of the hip and knee. He considers this a primitive type of reflex, as it is similar to that obtained in the case of monkeys. The infantile response persists until the child is able to walk, *i.e.*, until there is complete cerebral control over the movements of the lower limbs, when it is gradually replaced by the flexor response. In weakly, and more especially in ricketty children, the infantile response may persist up to the age of four years.

[7] Birm. Med. Rev., July, 1899. [8] Annals of Surgery, June, 1899. [9] Clin. Jour., June 7, 1899. [10] Arch. of Kinderheilk, B. xxv., H. iii. and iv. [11] Bost. Med. and Surg. Jour., June 15, 1899. [12] Med. Rec., April 15, 1899. [13] Brain, Spring Number, 1899.

